# Differential Salt Tolerance Strategies in Three Halophytes from the Same Ecological Habitat: Augmentation of Antioxidant Enzymes and Compounds

**DOI:** 10.3390/plants10061100

**Published:** 2021-05-30

**Authors:** AbdEl-Mageed F. M. Ghanem, Elsayed Mohamed, Ahmed M. M. A. Kasem, Abbas A. El-Ghamery

**Affiliations:** 1 Botany and Microbiology Department, Faculty of Science, Al-Azhar University, Assuit 71524, Egypt; AbdEl-MageedEldeeb.42@azhar.edu.eg (A.F.M.G.); amkasem@azhar.edu.eg (A.M.M.A.K.); 2 Botany & Microbiology Department, Faculty of Science (Cairo), Al-Azhar University, Madinat Nasr, Cairo 11751, Egypt; a.el_ghamery@azhar.edu.eg

**Keywords:** halophytes, Amaranthaceae, salinity, antioxidant enzymes, phenolic compounds

## Abstract

Understanding the salt tolerance mechanism in obligate halophytes provides valuable information for conservation and re-habitation of saline areas. Here, we investigated the responses of three obligate halophytes namely *Arthrocnemum macrostachyum*, *Sarcocornia fruticosa* and *Salicornia europaea* to salt stress (0, 100, 200, 400 and 600 mM NaCl) during their vegetative growth with regard to biomass, ions contents (Na^+^, K^+^ and Ca^2+^), chlorophyll contents, carotenoids, phenolic compounds, flavonoids, and superoxide dismutase, peroxidase and esterase activities. *S. europaea* showed the lowest biomass, root K^+^ content, Chl a/b ratio, and carotenoids under salinity. This reduction of biomass is concomitant with the increase in proline contents and peroxidase activity. On the other hand, the promotion of growth under low salinity and maintenance under high salinity (200 and 400 Mm NaCl) in *A. Macrostachyum* and *S. fruticosa* are accompanied by an increase in Chl a/b ratio, carotenoids, phenolics contents, and esterase activity. Proline content was decreased under high salinity (400 and 600 mM NaCl) in both species compared to *S. europaea*, while peroxidase showed the lowest activity in both plants under all salt levels except under 600 mM NaCl in *Arthrocnemum macrostachyum* compared to *S. europaea*. These results suggest two differential strategies; (1) the salt tolerance is due to activation of antioxidant enzymes and biosynthesis of proline in *S. europaea*, (2) the salt tolerance in *A. macrostachyum*, *S. fruticosa* are due to rearrangement of chlorophyll ratio and biosynthesis of antioxidant compounds (carotenoids, phenolics and flavonoids) which their cost seem to need less energy than activation of antioxidant enzymes. The differential behavior in halophytes of the same habitat confirms that the tolerance mechanism in halophytes is species-specific which provides new insight about the restoration strategy of saline areas.

## 1. Introduction

Soil salinization is a critical problem which influences agricultural activities and inhibits crop productivity. The food and agriculture organization (FAO) [1] reported approximately 831 million hectares (6% of total world land) were affected by salinity. Also, A high percentage of cultivated land around the world (more than 20%) is affected by salinity, and this percentage is daily increasing [2,3]. On the same side, population density, unfavorable environmental conditions and climate changes lead to reduce in cultivated lands [4]. Crop production decreasing with increasing population density could lead to famine around the world. Molecular biology and genetic engineering are powerful tools in the breeding of salt-tolerant crops. However, both approaches are slow, costly and they sometime fail to achieve the goal. Therefore, the cultivation of natural salt-tolerant plants as saline crops represents an easy and cheap solution for salt-affected areas [5,6].

Halophytes are plants that can maintain their biological activities and grow in salinity-affected soils [7]. One of the most popular effects of salinity is oxidative damage through the over generation of reactive oxygen species such as hydroxyl, superoxide and hydrogen peroxide [8]. Several morphological, physiological, biochemical and molecular changes have been observed to help halophytic plants to adapt to salinity [9,10,11,12]. These strategies depend on; maintaining the photosynthetic system through chlorophyll synthesis [13,14,15]; carotenoids enhancement or inhibition [16,17]; reactive oxygen species (ROS) production [8,18,19,20,21]; enzymatic antioxidant activation, such as superoxide dismutase (SOD) [20,22,23,24]; peroxidase [25] and Catalases [26,27]; non-enzymatic antioxidant synthesis, such as phenolic compound [28,29,30,31,32] and Flavonoids and [33]; osmoregulatory and compatible solutes synthesis [34], such as proline [35].

Amaranthaceae is a family of angiosperms which comprises about 165 genera and 2040 species [36] with a high number of xerohalophytes and halophytes around the world [37,38,39], 34 halophytic taxa are belonging to the family Chenopodiaceae/Amaranthaceae with a percentage of 22.08% of all halophytic angiosperms [40]. Among these taxa, *Arthrocnemum macrostachyum, Sarcocornia fruticosa* and *Salicornia europaea* are three halophytic plants distribute in the Mediterranean region [41]. *Arthrocnemum macrostachyum* is a perennial small shrub, erect to ascending stem, woody old stem and fleshy young stem, 30–40 cm in tall, like spike inflorescence, and papillose seeds. *Sarcocornia fruticosa* is a perennial sub shrub, erect to ascending stem, 20–80 cm in tall, and grey seeds covered with conical protuberances. *Salicornia europaea* is an erect annual herb with a cup-shaped branched stem, seed with conical protuberances [42]. These plants are considered cash crops due to their nutritional value and ecological importance in the phytoremediation of metals [43,44,45].

Fully understanding of salt tolerance mechanisms represents principle means in the management of the saline area and breeding of salt-tolerant cash crops [46,47]. Salt tolerance level is species-specific and the plant habitat contributes to the degree of salt tolerance and strategy among populations of the same species [24,48]. Mohamed et al. [22,23] reported that the Egyptian population of *Suaeda maritima* (Chenopodiaceae) has more salt tolerance than the Japanese population. Therefore, Egyptian Chenopodiaceae represents a unique genetic resource for saline agriculture application. To obtain more in-depth knowledge about the salt tolerance strategies of Chenopodiaceae, we hypothesized that Egyptian populations have unique salt tolerance levels and habitat of Mediterranean Sea influences on salt tolerance strategies of different species in this family. Our work aims to explore the salt tolerance strategies of three Egyptian Chenopods (Currently belong to Amaranthaceae) namely: *Arthrocnemum macrostachyum, Sarcocornia fruticosa* and *Salicornia europaea* from Damietta coast, through studying the effect of salt stress (0–600 Mm NaCl) on the growth parameters, chlorophyll contents, phenolic compounds, flavonoids, proline, malondialdehyde (MDA), esterase and antioxidant enzymes (superoxide dismutase, catalase and peroxidase activities).

## 2. Results

### 2.1. Effect of Salinity on Na^+^, K^+^ and Ca^2+^ Contents

While, K^+^ content in the shoot system was slightly decreased at all saline concentrations except at 100 mM NaCl and the root system K^+^ content was increased at all salt concentrations. The Na^+^ and Ca^2+^ shoot and root contents were gradually increased by increasing salt concentrations in *A. macrostachyum*. In the case of *S. europaea*; shoot Na^+^ and Ca^2+^ were increased at all concentrations, while K^+^ content increased at 100 and 200 mM NaCl only. In the root system, Na^+^ content increased in all concentrations, Ca^2+^ increased at 600 mM NaCl, but K^+^ decreased at all concentrations. In *S. fruticosa* Na^+^, Ca^2+^ and K^+^ content decreased at all concentrations in the shoot system except at 600 mM NaCl, both Na^+^ and Ca^2+^ were increased with respect to control. On the other hand, root Na^+^ and Ca^2+^ contents increased with salinity and K^+^ increased only at 200 and 400 mM NaCl (Table 1).

### 2.2. Effect of Salinity on Growth Parameters

#### 2.2.1. Effect of Salinity on Biomass Production

Two-way ANOVA analysis for studied plants showed significant effects for the plant and species, and their interactions (*p* < 0.001) for all parameters (Table 2). These interactions support the different responses of the species to salinity.

**Figure 1:**
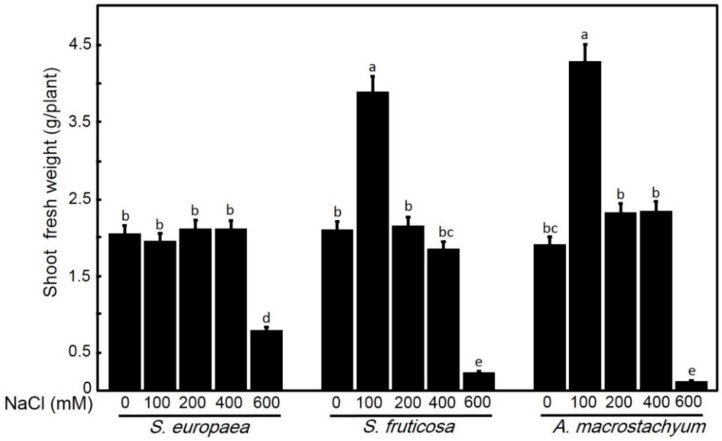
Shoot fresh weights of *S. europaea, S. fruticosa* and *A. macrostachyum* under different NaCl concentrations. Mean ± SE of three replicates. Different letters indicate significant differences (*p* < 0.05).

**Figure 2:**
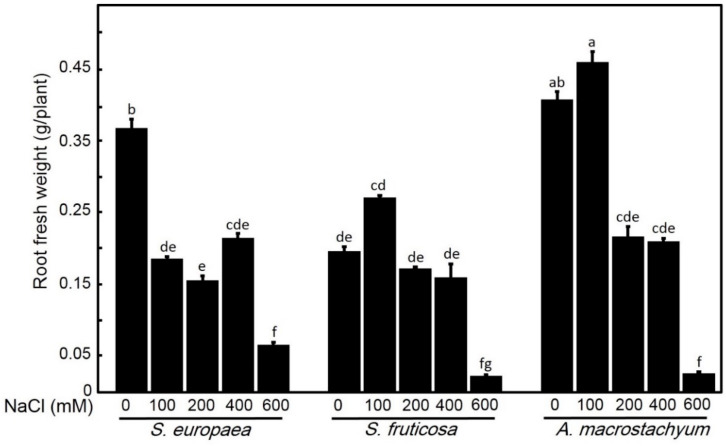
Root fresh weights of *S. europaea, S. fruticosa* and *A. macrostachyum* under different NaCl concentrations. Mean ± SE of three replicates. Different letters indicate significant differences (*p* < 0.05).

**Figure 3:**
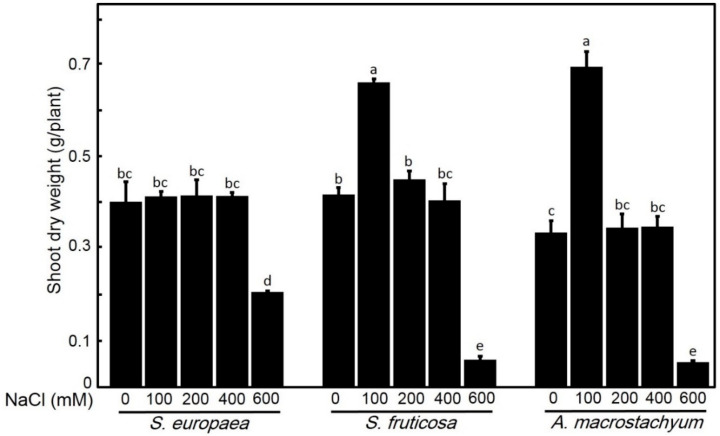
Shoot dry weights of *S. europaea, S. fruticosa* and *A. macrostachyum* under different NaCl concentrations. Mean ± SE of three replicates. Different letters indicate significant differences (*p* < 0.05).

**Figure 4:**
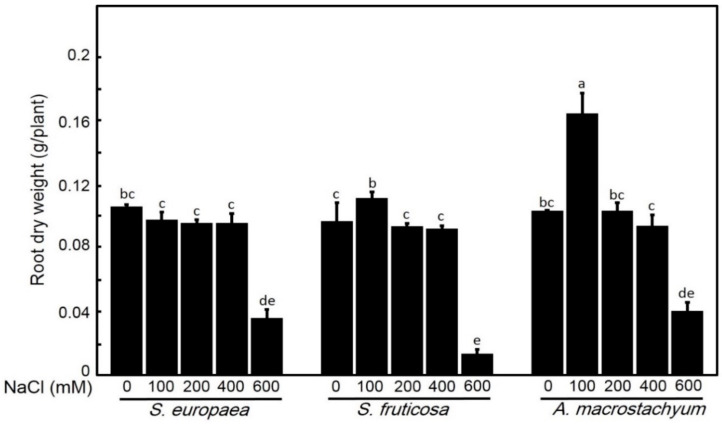
Root dry weights of *S. europaea, S. fruticosa* and *A. macrostachyum* under different NaCl concentrations. Mean ± SE of three replicates. Different letters indicate significant differences (*p* < 0.05).

One-way ANOVA showed that each species has its response for different parameters at applied saline concentrations. *A. macrostachyum* and *S. fruticosa* showed highest shoot and root fresh and dry weights with significant increasing at 100 mM NaCl and slightly increasing at 200 and 400 mM NaCl. At 600 mM NaCl, both species showed significant decreases in these parameters. In contrast, *S. europaea* showed non-significant difference in shoot fresh and dry weights and root dry weight at low and moderate salt treatments, but root fresh weight showed a significant decrease at all treatments, and all parameters were highly decreased at 600 mM NaCl (Figure 1, Figure 2, Figure 3 and Figure 4).

#### 2.2.2. Effect of Salinity on Chlorophyll Contents

**Figure 5:**
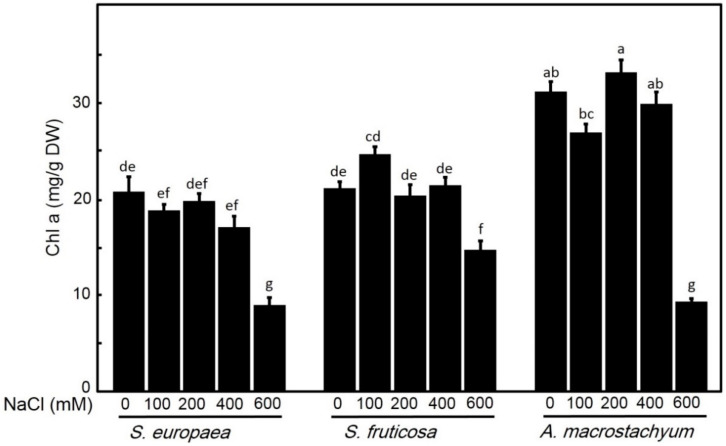
Chlorophyll a contents of *S. europaea, S. fruticosa* and *A. macrostachyum* under different NaCl concentrations. Mean ± SE of three replicates. Different letters indicate significant differences (*p* < 0.05).

**Figure 6:**
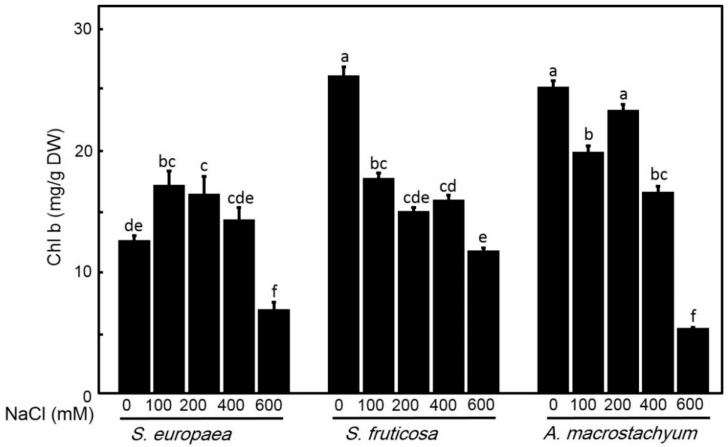
Chlorophyll b contents of *S. europaea, S. fruticosa* and *A. macrostachyum* under different NaCl concentrations. Mean ± SE of three replicates. Different letters indicate significant differences (*p* < 0.05).

**Figure 7:**
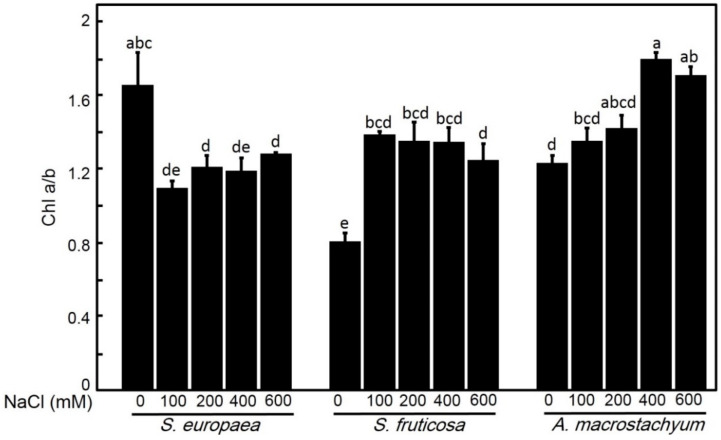
Chlorophyll a/b ratios of *S. europaea, S. fruticosa* and *A. macrostachyum* under different NaCl concentrations. Mean ± SE of three replicates. Different letters indicate significant differences (*p* < 0.05).

Chlorophyll contents showed different responses to salt treatments in all studied species (Figure 5 and Figure 6). Chlorophyll a contents showed non-significant differences in *A. macrostachyum* and *S. fruticosa* with slightly increasing at 200 mM NaCl in *A. macrostachyum* and at 100 mM in *S. fruticosa*, and it was significantly decreased at 600 mM NaCl in both species. In contrast, *S. europaea* showed slightly non-significant decreases at low and moderate treatments and significantly decreasing at higher concentrations. For chlorophyll b, while *A. macrostachyum* showed non-significant differences at low and moderate NaCl concentrations and significant decreases at 400 and 600 mM NaCl, *S. fruticosa* showed significant decreases at all salt treatments. In the case of *S. europaea,* chlorophyll b contents were significantly increased at low and moderate concentrations NaCl and significantly decreased at high NaCl concentrations. For chlorophyll a/b ratio, it was significantly increased at high salt concentrations in *A. macrostachyum*; and at all salt levels in *S. fruticosa*, and significantly decreased at all salt concentrations in *S. europaea* (Figure 7).

#### 2.2.3. Effect of Salinity on Carotenoids

**Figure 8:**
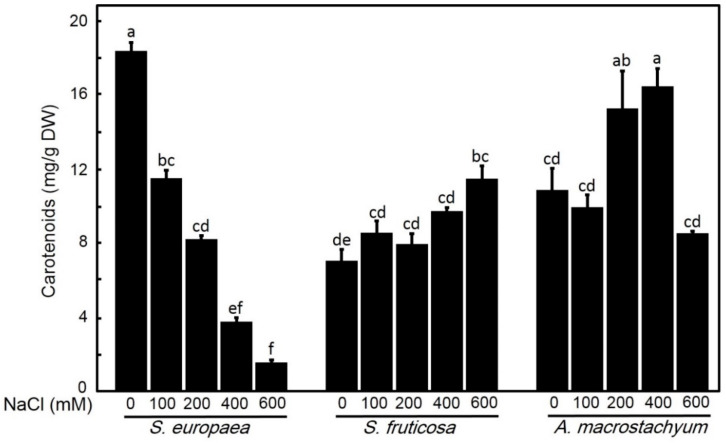
Carotenoids contents of *S. europaea, S. fruticosa* and *A. macrostachyum* under different NaCl concentrations. Mean ± SE of three replicates. Different letters indicate significant differences (*p* < 0.05).

Carotenoids concentration showed significant increases with saline concentrations except at 100 and 600 mM NaCl which show non-significant differences in respect to control in *A. macrostachyum* while *S. fruticosa* showed non-significant increases under low and moderate salt concentrations, and a significant increase under high salt level. In contrast, *S. europaea*, showed significant decreases with increasing NaCl concentration (Figure 8).

#### 2.2.4. Effect of Salinity on Total Phenolic Contents

**Figure 9:**
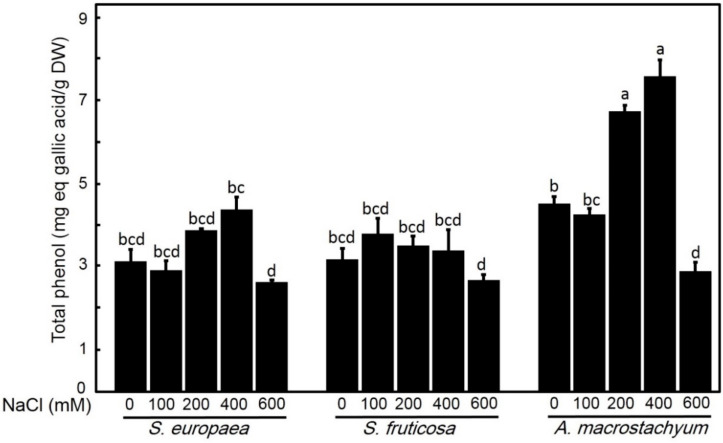
Total phenol contents of *S. europaea, S. fruticosa* and *A. macrostachyum* under different NaCl concentrations. Mean ± SE of three replicates. Different letters indicate significant differences (*p* < 0.05).

Phenolic compound contents in *A. macrostachyum* significantly increased at moderate and high salinity levels (200 and 400 mM NaCl) with a slightly non-significant difference at 100 mM NaCl. In *S. fruticosa* and *S. europaea* slightly non-significant increases in phenolic contents were recorded at all saline concentrations. Interestingly, the three species showed significant decreases at 600 mM NaCl (Figure 9).

#### 2.2.5. Effect of Salinity on Flavonoid Contents

**Figure 10:**
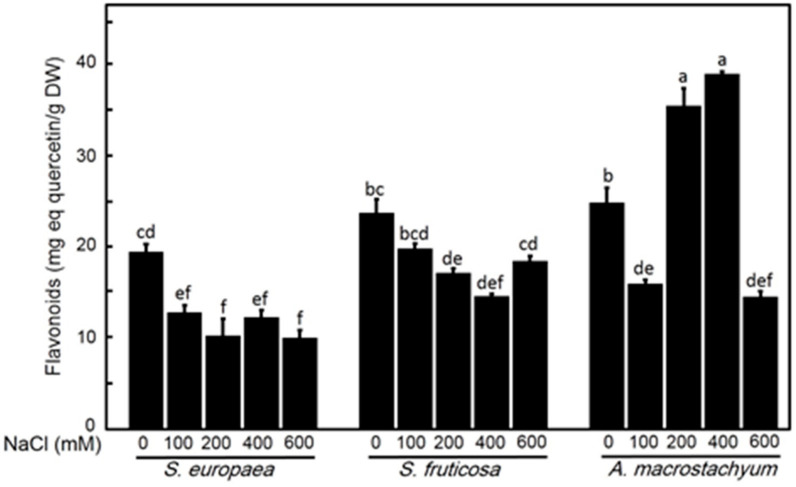
Total flavonoids contents of *S. europaea, S. fruticosa* and *A. macrostachyum* under different NaCl concentrations. Mean ± SE of three replicates. Different letters indicate significant differences (*p* < 0.05).

*A. macrostachyum* showed significant increases in flavonoid contents at all treatments but significantly decreased at 100 and 600 mM NaCl. In contrast, *S. fruticosa* and *S. europaea* showed significant decreases in flavonoid contents with a slightly non-significant difference at 600 mM NaCl in *S. fruticosa* (Figure 10).

#### 2.2.6. Effect of Salinity on Total Malondialdehyde (MDA) Content

**Figure 11:**
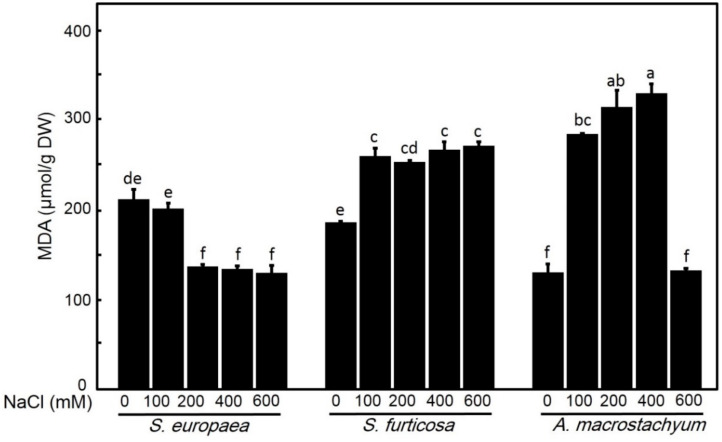
Malondialdehyde contents of *S. europaea, S. fruticosa* and *A. macrostachyum* under different NaCl concentrations. Mean ± SE of three replicates. Different letters indicate significant differences (*p* < 0.05).

*A. macrostachyum* and *S. fruticosa* showed significant increases in MDA concentrations in all treatments except at 600 mM NaCl, which observed a non-significant difference compared to control. *S. europaea* showed significant decreases at all salt concentrations except at 100 mM NaCl, which showed a non-significant difference in respect to control (Figure 11).

#### 2.2.7. Effect of Salinity on Proline Content

**Figure 12:**
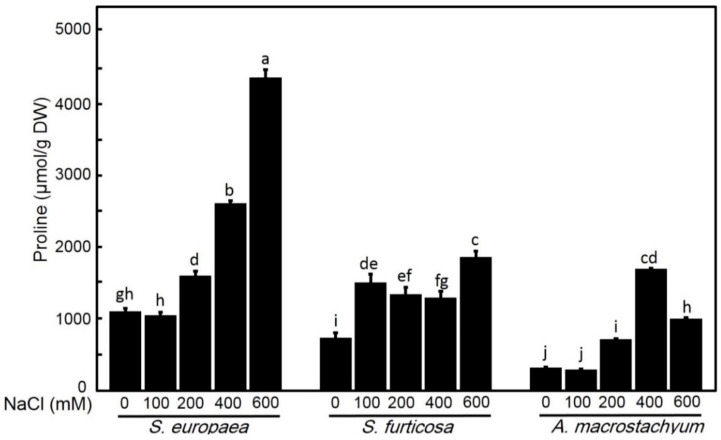
Proline contents of *S. europaea, S. fruticosa* and *A. macrostachyum under* different NaCl concentrations. Mean ± SE of three replicates. Different letters indicate significant differences (*p* < 0.05).

While *S. fruticosa* showed significant increases in proline content with increasing salt concentrations, Proline content in *A. macrostachyum* and *S. europaea* were significantly increased at 200, 400 and 600 mM NaCl only, and higher values of proline in *S. europaea* were recorded at 600 mM NaCl in respect to the other two species (Figure 12).

### 2.3. Isozymes Analysis

#### 2.3.1. Esterases

**Figure 13:**
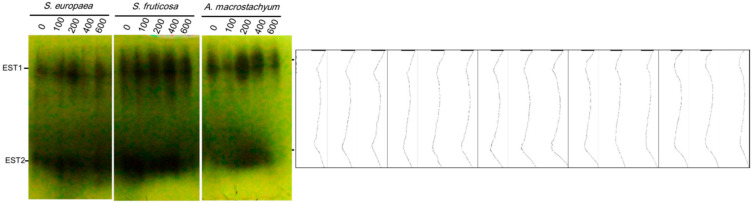
Esterase isozymes of *S. europaea, S. fruticosa* and *A. macrostachyum* under different NaCl concentrations.

The electrophoretic analysis using native PAGE showed two esterase loci in all studied species and under all treatments with different amounts and intensities. The highest intensities were observed in *A. macrostachyum* and *S. fruticosa* at 200 and 400 mM NaCl and 100 and 200 mM NaCl in *S. europaea* (Figure 13).

#### 2.3.2. SOD Isozymes

SOD activity was increased at high salt levels in *A. macrostachyum* and *S. europaea*, and under low and moderate salinity in *S. fruticosa* (Supplementary S1).

#### 2.3.3. POD Isozymes

**Figure 14:**
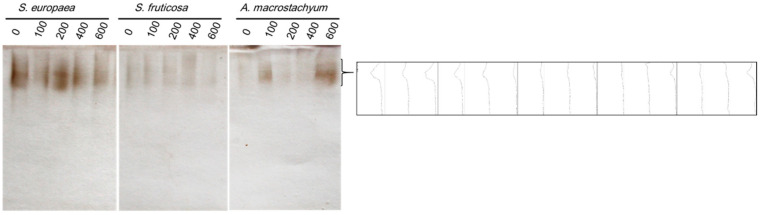
Peroxidase isozymes of *S. europaea, S. fruticosa* and *A. macrostachyum* under different NaCl concentrations.

POD enzyme showed a unique locus in all studied plants and at all treatments. The highest intensities were recorded at 100 and 600 mM NaCl in *A. macrostachyum*, and at 200 mM NaCl in *S. europaea* which showed the highest POD activity in respect to the other two species. On the other hand, weak activity was observed at all treatments in *S. fruticosa* (Figure 14).

Pearson correlation and principal component analysis.

**Figure 15:**
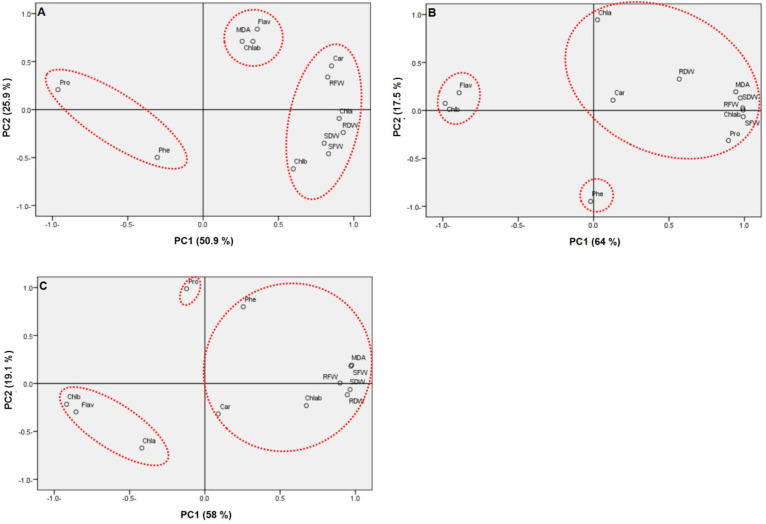
Principal components analysis bi-plot. Values of the studied parameters were analyzed under salinity (100 mM NaCl) with respect to control. Growth parameters (shoot fresh weight: SFW, root fresh weight: RFW, shoot dry weight: SDW, root dry weight: RDW), chlorophyll a: Chl a, chlorophyll b: Chl b, Chl a/b, carotenoids: Car, Proline: pro, malondialdehyde: MDA, phenolics: Phe, Flavonoids: Flav. In (**A**) *Salicornia europaea,* (**B**) *Sarcocornia fruticosa*, and (**C**) *Arthrocnemum macrostachyum*.

For *Salicornia europaea*, growth parameters have positive correlations under salinity with Chl a, Chl b, and carotenoids, and negatively correlated with proline content (Table S1). In the same context, under 100 mM saline treatment, principal component analysis showed PC1 and PC2 described 50.9%, and 25.9% of the variance, respectively (Figure 15). Three groups were observed from this analysis; Growth parameters (shoot fresh weight, shoot dry weight. root fresh weight and root dry weight), Chl a, Chl b and carotenoids constructed the first group, Flavonoids, Chl a/b, MDA formed the second group, and both proline and phenolic compound represented the third group. For *Sarcocornia fruticosa*, growth parameters were positively correlated with Chl a, Chl b and Chl a/b but negatively correlated with carotenoids, MDA, phenolic compounds and flavonoids (Table S1). In contrast, PC1 and PC2 explained 64%, and 17.5% of the variance, respectively. Three groups were also visualized from this analysis; Growth parameters, Chl a, Chl a/b, MDA and proline formed the first group, Chl b and flavonoids represented the second group, and phenolics represented the third group. For *Arthrocnemum macrostachyum*, Our results showed significant negative correlations between most growth parameters with carotenoids, proline, flavonoids and phenolic compounds, Chl a/b and MDA, but positively correlated with Chl b (Table S1). On the other hand, Principal component analysis showed PC1 and PC2 described 58%, and 19.1% of the variance, respectively. Three groups were also noticed from PCA analysis; Growth parameters, carotenoids, phenolic compounds, Chl a/b and MDA formed the first group, Chl a, Chl b and flavonoids constructed the second group, and proline formed the third group.

## 3. Discussion

Exploration of salt tolerance mechanisms of many halophytes species is of considerable value for the selection of suitable crops for saline agriculture. In this study, three halophytic species *Arthrocnemum macrostachyum*, *Sarcocornia fruticosa* and *Salicornia europaea* (Amaranthaceae/Chenopodiaceae) were collected from the same saline habitat and tested for their tolerances to salinity.

For growth criteria, *A. macrostachyum* and *S. fruticosa* improved their fresh and dry weight when grown under low and moderate salt concentrations, but their fresh weights were reduced at high salinity. *A. macrostachyum* had the optimum growth at 400 mM NaCl and its growth decreased at 600–1000 mM NaCl [49]. The same induction trend of growth under moderate salinity (170–510 mM NaCl) was observed in *A. macrostachyum* and *S. fruticosa* from Spain, with a decline trend under high salt conditions [14,50]. Also, García-Caparrós et al. [51] reported that total dry weight and relative growth rate of *S. fruticosa* decreased significantly under low and moderate salinity (100 and 200 mM NaCl) for 60 days. Therefore, our results suggest that Egyptian *A. Macrostachyum* and *S. fruticosa* need low salt levels for optimal growth, and they could maintain their growth under moderate and high salinity (200 and 400 Mm NaCl). The variation in salt tolerances of both plants in the previous studies might be because of the maternal habitats of these populations. Mohamed et al. [24] reported that maternal salinity plays important role in salt tolerance during the growth of *Zygophyllum ccocenium.*

On the other hand, *S. europaea* showed significant decreases in shoot fresh and dry weights at high salinity levels but slightly non-significant variation under moderate salinity. Ungar et al. [52] reported that *S. europaea* growth was increased under moderate salinity (170–510 mM NaCl). In contrast, *S. rubra* had the optimal growth in the absence of salt to 200 mM NaCl while its growth was inhibited with further increase of salt level. The decline of root biomass under moderate salinity suggests the severe effect of salinity on the root system than shoot and the adaptive strategy to avoid more uptakes of toxic ions [53].

Inorganic ions play role in maintain osmotic and turgor pressure in halophytes more than glycophytes, which predominantly depend on the increased synthesis of de novo compatible solutes [54]. Flowers et al. [55] reported that the Na^+^ is one of the most important ions which play important role in adjusting cellular osmotic potential. Our results showed that *A. macrostachyum* and *S. europaea* Na^+^ contents increased with increasing external NaCl concentrations while Na^+^ content in *S. fruticosa* was only increased under high salinity. This increase in Na^+^ has a role in maintain shoot osmotic and turgor. Redondo-Gomez et al. [14] and Khan et al. [56] reported increasing Na^+^ content with increasing external NaCl concentrations in *A. macrostachyum* because halophytes have a unique ability for osmotic adjustment due to accumulation of Na^+^ in vacuoles, and K^+^ and organic solutes in the cytosol [57,58]. The stimulation of K^+^ in halophytes root under saline conditions is well documented in many plants, such as *Suaeda monoica* and *Triglochin maritima* [59]. In the present study, while K^+^ ions in roots were increased with increasing salinity in *A. macrostachyum* and *S. fruticosa* and declined in *S. europaea*, Shoot K^+^ ions increased at low concentration and decreased at high and moderate concentrations. These results suggest that K^+^ content can be used as a marker for discrimination between salt tolerance strategies in halophytes [58]. In the same context, Ca^2+^ increased with salinity, This increase is due to its vital role in salt adaptation through binding of Ca^2+^ with SOS_3_ and subsequently activate SOS_2,_ this complex stimulates Na^+^/ H^+^ antiporter which plays a crucial role in the regulation of Na^+^ ions in the cytosol [60].

In saline habits, soil salinity and arid climate greatly affect the synthesis of pigments in plants [61] and salinity reduces the net photosynthetic rate [62]. Redondo-Gómez et al. [14] reported that *A. macrostachyum* can improve or adjust the rate of photosynthesis under saline conditions. Aghaleh et al. [17] and Akcin and Yalcin [63] reported that photosynthetic pigments of *S. europaea* from Iran and Turkey were affected by increasing soil salinity. Our data showed non-significant variations in chl a under all salinity levels except in *S. europaea* under very high salinity (600 Mm NaCl), and significant decreases of chl b were only observed in *S. fruticosa* under all saline concentration and in *A*. *macrostachyum* and *S. europaea* under high saline concentration (600 Mm NaCl). This result suggests that *S. fruticosa* has a differential response to salt stress compared to *A. macrostachyum* and *S. europaea.* The increase in Chl a/b ratio in *A. macrostachyum* and *S. fruticosa* suggests that both species had more adaptation to saline conditions than *S. europaea* [24,64].

Carotenoids play a vital role as non-enzymatic antioxidants in protecting photosynthetic system. Our results showed significant increases in carotenoids with the elevation of NaCl concentration in *A. macrostachyum* and *S. fruticosa,* except at low salinity level for both species and under 600 in *A. macrostachyum*. This increase in carotenoid concentration may be one strategy to maintain chlorophyll amount and not decreasing it with different salinity concentrations. A similar study confirmed the increase in carotenoids in *Nitraria retusa* was associated with increasing salt tolerance [65]. In contrast, Carotenoids in *S. europaea* decreased significantly at all treatments. Such decreases in carotenoid contents under salinity stress were reported in different plant species [17,61,63,66]. These results suggest that carotenoids play an important role in the salt tolerance of *A. macrostachyum* and *S. fruticosa* than in *S. europaea.*

Phenolic compounds are secondary metabolites that play an important role in protecting plants against oxidative stress [67]. Increasing phenolic compounds synthesis is considered one of the most important methods in water deficiency resistance [68]. The synchronous significant increase of phenolic compounds and flavonoids in *A. macrostachyum* at moderate and high salinity levels indicates the importance of these compounds in stress tolerance in a synergistic relationship with carotenoids that also showed significant increases with salinity. Król et al. [69] and Caliskan et al. [70] reported that the metabolism of phenylpropanoid and phenolic compounds accumulation were enhanced in different plant species in response to different environmental stress conditions. Along the same line, the non-significant decrease in chlorophyll content in *A. macrostachyum* at high salinity level is due to increase in phenolic compounds contents at the same salinity level. This was supported by the finding of Bhattacharya et al. [71] who reported that phenolic compounds play a vital role in the biosynthesis of lignin and pigments in plants. Also, *S. fruticosa* showed a slight increase in phenolic contents at moderate NaCl concentrations and non-significant differences at other concentrations. This indicates that moderate salinity stimulates the production of phenolic compounds in *S. fruticosa.* On other hand, a constant or slight increase in total phenolic contents in *S. europaea* at different salinity levels was associated with the decrease in carotenoids. These results may indicate the importance of phenolic compounds in the alleviation of deleterious effects of salt stress in *A. macrostachyum* and *S. fruticosa* [72,73,74,75].

*A. macrostachyum* showed significant increases in flavonoid contents at all treatments except at low salinity which had a significant decrease. In contrast, *S. fruticosa* and *S. europaea* showed significant decreases in flavonoid contents but not at high salinity level in *S. fruticosa* which showed a slightly non-significant decrease. Brown et al. [76] reported that flavonoids act as auxin transport inhibitors, therefore, high promotion of shoot growth under low salinity (100 Mm NaCl) in *A. macrostachyum* and *S. fruticosa* may be due to low flavonoids content. The positive performance of shoot growth, despite its low root biomass, may be due to the same previous reason.

Malondialdehyde (MDA) concentration expresses the extent of destruction in the membrane because it acts as a common end product of lipid peroxidation [19]. Jithesh et al. [77] and Mohamed et al. [23] reported the presence of a positive correlation between salinity stress and MDA content in halophytic plants. *A. macrostachyum* and *S. fruticosa* showed significant increases in MDA concentration in all treatments except at 600 mM NaCl in *A. macrostachyum*. This result is in agreement with Abd El-Maboud [75] who reported increasing in MDA concentration in *A. macrostachyum* in the summer season. On other hand, *S. europaea* showed a significant decrease in MDA content with no effect at low salinity concentrations. This result contradicts the reported increase in MDA in *S. europaea* collected from Iran with the increase in salinity level [17]. This decrease in MDA concentration in *S. europaea* may be due to an increase in peroxidase activity, which was often stored at the cytosol, peroxisome and vacuole [78,79]. The increasing of peroxidase activity plays an active role in free radical oxidative stress inhibition, which leads to protect the membrane and decrease lipid peroxidation. Also, decreasing MDA may be due to the increasing accumulation of proline content in *S. europaea* than the other two species, which act as non-enzymatic antioxidant enzymes and this suggestion is in agreement with proline having a role in ROS scavenger [80].

Proline accumulates in the cell as an osmoregulatory solution which plays an important role in the adaptation of halophytes to high salinity levels [81]. Increasing in the accumulation of proline in response to salinity stress was reported in different plant species by different researchers [82,83]. Increasing proline synthesis helps in decreasing water loss and ions’ toxicity [84]. Our results showed a significant increase in proline contents at a high salinity level in all studied species. This increase may indicate upregulation of proline synthesis [85]. Increasing proline content in *S. europaea* at high salinity than the other two species may be important to compensate for the decrease in the carotenoids and flavonoids contents and to help as free radical scavengers.

Pectin is one of the basic components of a plant cell wall. It can be both methyl-esterified and acetyl-esterified. De-esterification occurs by specific esterases [86]. Esterase plays a vital role in avoiding the salt-induced imbalance in cell wall formation. Our results showed two esterase loci in all studied species and under all treatments with the higher intensities in *A. macrostachyum* and *S. fruticosa* at moderate salinity level, and at low and moderate salinity levels in *S. europaea*. These results are in agreement with Dasgupta et al. [87] who reported that esterase isoforms intensities were increased with elevating salt concentration. Mohamed et al. [25] found esterase has two isoforms in *Pancratium maritimum* and their intensities were increased under moderate saline concentration.

For *S. europaea* under salt stress (100 mM NaCl), Principal component analysis observed the arrangement of growth parameters, chlorophyll parameters, MDA and flavonoids on the positive X-axis, while proline and phenolic compounds grouped on the negative X-axis. This result suggests the salt tolerance of this species due to the accumulation of proline and phenolic compounds. In contrast, all parameters grouped on the positive X-axis, except Chl b and flavonoids were observed on the negative X-axis for *S. fruticosa,* and Ch b, flavonoids, Chl a and proline grouped on the negative axis for *A. macrostachyum*. These results confirm the growth promotion of both species due to increasing of Chl a/b ratio and the decline of flavonoids contents.

The promotion of growth parameters in *S. europaea* under 600 Mm NaCl compared to *A. macrostachyum* and *S. fruticosa* may be due to the decline of flavonoids accumulation in *Salicornia* under this salt level compared to the other two species. The decline in most parameters under 600 mM NaCl in *A. macrostachyum* suggests the deleterious effects of this concentration on this species.

Superoxide dismutase is considered the most important enzyme during the growth of plants under biotic and abiotic stress through catalyzing the dismutation of superoxide radicals into H_2_O and Oxygen [88,89,90]. Nisar et al. [91] reported constitutive and decline of SOD activity in germinating black and brown *A. macrostachyum* seeds respectively under salinity. On the other hand, salinity induced promotion in SOD activity in *S. europaea* seedlings [92]. The induction of SOD under salinity was a prominent feature in halophytes such as *Suaeda maritima, Pancratium maritimum and Zygophyllum coccenium* [22,23,24,25]. In this study, SOD activity increased under high salinity in *A. macrostachyum* and *S. europaea*, and under moderate salinity in *S. fruticosa*. These results suggest a differential mechanism for SOD under salinity in these species.

POD enzyme has a major protective role for the cell against hydrogen peroxide which is produced under stress conditions [93,94]. Our study showed that POX enzyme has a stable faint locus at all salinity levels in *S. fruticosa*, and at high and moderate salinity in *A. macrostachyum* and *S. europaea*. The highest POD activity was recorded in *S. europaea* in respect to other species. This increase in both peroxidase and SOD activities under higher salinity may decrease free radical concentrations and protects membranes from lipid peroxidation, and hence the low MDA concentration in *S. europaea* than the other two species. Also, this indicates that POD is one of the most important strategies in salt tolerance in *S. europaea* more than the other two species.

From the foregoing discussion, the three halophytic species that are belonging to the same family and collected from the same saline ecological habitat showed differential mechanism to salt tolerance. The salt tolerance of *S. europaea* is derived from the promotion of proline level and peroxidase activity. The stable shoot and decline in root biomass suggest investment of energy in the promotion of antioxidant enzymes and compounds than use it in the growth process. This was supported statistically by the presence of a significant negative correlation between growth parameters and proline contents. In contrast, salt tolerance of *A. macrostachyum and S. fruticosa* is concomitant with rearrangement of chlorophyll contents, high level of carotenoids and phenolic compounds, and activation of esterase enzyme. This conclusion seems to be confirmed by the negative correlation between most of these compounds and the growth parameters of both species. The positive performance of both species’ biomass, compared to *S. europaea,* suggests little energy was used in the salt tolerance mechanism in these plants. Also, a trade-off strategy between the growth process and defense system was noticed in the case of *S. europaea*. These results confirm differential salt tolerance strategies of different halophytes in the same habitat which provide valuable information in the selection of the best strategy in re-habitation of saline coastal areas.

## 4. Materials and Methods

### 4.1. Plant Seeds Collection

Inflorescences containing mature dry seeds of three species belong to Amaranthaceae family were collected from a halophytic region Damietta–Alexandria road during June 2018 and transported to the laboratory. Seeds were manually separated from the inflorescence and stored in paper bags until use. Studied species soil analysis was conducted according to Jackson [95]. The soil electrical conductivity was 15.325 ds/m and pH values were 9.36, Ca, Mg, Cl and HCO_3_ concentrations were 0.035%, 0.01%, 0.4686% and 0.03355% respectively.

### 4.2. Growth Conditions

Seeds of studied plant species were surfaced sterilized using 70% ethyl alcohol for 30 s followed by 3.5% (*v*/*v*) Sodium hypochlorite for 5 min, then washed thoroughly with distilled water [22]. Sterilized seeds of each species were sown in 25 replicates plastic pots with 20 cm height and 10 cm in diameter containing sandy soil and irrigated with 150 mL of 20% MS medium. The germination was carried out under natural greenhouse conditions (temperature range 14–28 °C, humidity about 40%, and photoperiod 14: 10 light: dark) for 30 days. After this period, 15 plastic pots of each species with uniform seedlings size were chosen and divided to five groups; each group contains three replicates, and each replicate containing five plants. Five treatments were used in this experiment (0, 100, 200, 400, and 600 mM NaCl) and plants were irrigated with 1 L of 20% MS medium prepared in distilled water, 100, 200, 400 and 600 mM NaCl (150 mL weekly) for two months.

### 4.3. Determination of Na^+^, K^+^ and Ca^2+^

Air-dried shoot and root were grounded to fine powders and 0.2 g of each sample were treated with 7:3 sulfuric: perchloric acid mixture. Cations’ concentrations were determined according to Jackson [95].

### 4.4. Growth Parameters

#### 4.4.1. Shoot and Root Fresh and Dry Weight Determination

For each treatment, five plants were used for the shoot and root fresh and dry weights determination. Plants were removed from the pots and washed under tap water to remove any dust then plants were dried using paper tissues. Aerial parts and root system were separated and weighed using sensitive balance, after that plants were dried using a hot air oven at 70 °C for 72 h until the weights become constant and reweighed to record dry weight.

#### 4.4.2. Determination of Photosynthetic Pigments

For the determination of chlorophyll a, b and carotenoids, 0.1 g of plant tissue was homogenized in 10 mL of 80% acetone then centrifuged at 5000 rpm for 10 min. Supernatant absorbance was read at 663, 645, and 470 nm and photosynthetic pigment contents were calculated from the equations as described by Lichtenthaler and Wellburn [96].

#### 4.4.3. Determination of Malondialdehyde (MDA) Content

Malondialdehyde (MDA) was determined according to Carmak and Horst [97] methods, 0.2 g of fresh plant aerial system were homogenized in 2 mL of 0.1% (*w*/*v*) trichloroacetic acid (TCA) at 4 °C. The homogenate was centrifuged for 10 min at 1000 rpm and to 0.5 mL of the supernatant, 3 mL of 0.5% (*v*/*v*) Thiobarbaturic acid (prepared in 20% TCA) was added. The mixture was incubated in 95 °C water bath with continuous shaking for 50 min, and then samples were placed in an ice bath until the temperature decreased to 25°C. The samples were re-centrifuged for 10 min at 10,000 rpm and the absorbance of the mixture was read at 532 nm. The non-specific absorption read at 600 nm was subtracted from all the readings and the MDA contents were calculated using the absorption coefficient as follows:

MDA level (nmol) = Δ (A 532 nm−A 600 nm)/1.56 × 10^5^ (1)

#### 4.4.4. Determination of Proline Content

Proline was determined using Bates et al. [98] method as follows; 0.5 g of fresh plant shoot were homogenized in 4 mL of 3.0% Sulphosalicylic acid. Then the homogenate was centrifuged for 10 min at 1000 rpm. To 1 mL of the supernatant 2 mL of acid Ninhydrin reagent and 2.0 mL of glacial acetic acid were added in a test tube, Then the mixture was incubated in a water bath at 100 °C for 60 min. then the mixture was cooled suddenly in an ice bath. After cooling, 4 mL of toluene were added to the solution mixture and vortex. The chromophore containing toluene (upper layer) was transferred to a new test tube. Finally, the absorbance was read at 520 nm using a spectrophotometer and Toluene as a blank. The concentration of proline was determined using the standard curve and expressed as mg g^−1^.

#### 4.4.5. Determination of Total Phenolic Compounds and Flavonoids

For the determination of phenolic compounds, 0.1 g of the shoot was homogenized in 10 mL of 70% acetone, then centrifuge at 5000 rpm for 10 min. To 1 mL of supernatant, 2 mL of sodium carbonate (15%) and 1 mL Folin–Ciocâlteu reagent (FCR) was added and the absorbance was recorded at 650 nm. Gallic acid was used as a standard for the determination of phenolic contents [99]. For total flavonoid, the aluminum trichloride method was used, to 1 mL of extract 2.5 mL of AlCl_3_ reagent in ethanol 90% (20.0 mg/mL), then incubated at room temperature for 40 min. and the absorbance was recorded at 415 nm. Quercetine was used as a standard for flavonoids determination [100]. All absorbances were determined using Jenway 7315 spectrophotometer, Jenway Scientific Instrumental Company, UK.

### 4.5. Isozymes Analysis

#### 4.5.1. Enzymes Extraction and Detection

For protein extraction; 0.2 g of plant aerial part tissue were macerated in 1 mL of 50 mM Tris HCl buffer (pH 6.8) containing 1 mM EDTA, 1 mM DDT, and 20 mg polyvinyl polypyrrolidone (PVPP) using chilled ceramic mortar and pestles. The homogenate was centrifuged at 10,000 rpm for 10 min at 4 °C. The supernatant was stored in 4 °C until used. The protein concentration was determined by spectrophotometry according to Lowry’s method [101] using bovine serum albumin as a standard.

Native discontinuous system was prepared according to Laemmli [102] without adding Sodium dodecyl sulfate (SDS) and 50 μg from each sample were loaded directly without denaturation. The running voltage was started at 80 V for 30 min then increased to 120 V until the loading dye migration reached the bottom of the resolving gel.

For visualization of esterases, the gel was incubated in 100 mL of 100 mM Sodium phosphate buffer (pH 7) containing 40 mg α-Naphthyl acetate and 0.2 g fast blue RR for 30 min. in dark at 37 °C, and then the gel was fixed in 7% acetic acid solution [103].

For visualization Peroxidase activity, Seevers et al. [104] method was used, after electrophoresis gel was incubating for 30 min at 25 °C in 200 mM Sodium acetate buffer (pH 5) containing 3% H_2_O_2_ and 1.3 mM Benzidine, and the gel was fixed in 30% fixing solution.

For visualization Superoxide Dismutase (SOD), the gel was incubated in 200 mM K-phosphate buffer (pH 7.8) containing 0.1 mM riboflavin and 0.24 mM Nitroblue tetrazolium for 30 min, and then the gel was stained by exposure to fluorescence light.

All gels were photographed using Cannon kiss4 digital camera then transferred to a computer and converted into density profile using Image J program [105].

#### 4.5.2. Statistical Analysis

All data were expressed as means with standard error, and Levene’s test was used to investigate the homogeneity of variances of all data, then the data were subjected to one-way ANOVA and Tukey test. Two-way ANOVA was applied to determine the effect of salinity, species, and their interaction with all parameters. Principal component analysis was used to explore the correlation between growth parameters and studied organic compounds under salinity (100 mM NaCl). Also, Pearson’s correlation coefficient was applied to investigate the correlation between all studied parameters under salinity treatments. All statistical analyses were carried out using SPSS 16.0 software. The means comparison was set at *p* < 0.05 and values denoted by the same letter are not significantly different.

## Figures and Tables

**Table 1:** Analysis of Na^+^, K^+^ and Ca^2+^ content (µmol/g DW) in root and shoot of namely *Salicornia europaea*, *Sarcocornia fruticosa* and *Arthrocnemum macrostachyum* under different salt concentrations. Different letters indicate significant differences.

species	NaCl (mM)	Shoot			Root		
		Na^+^ (µmol g^−1^ DW)	K^+^ (µmol g^−1^ DW)	Ca^2+^ (µmol g^−1^ DW)	Na^+^ (µmol g^−1^ DW)	K^+^ (µmol g^−1^ DW)	Ca^2+^ (µmol g^−1^ DW)
***S. europaea***	0	2217 ± 5 m	387 ± 4 ef	293 ± 8 j	406 ± 19 k	493 ± 10 c	58.75 ± 6 bc
	100	8239 ± 12 cd	607 ± 11 a	2166 ± 22 b	985± 9 h	301 ± 13 g	39 ± 3 ef
	200	8637 ± 10 c	543 ± 11 b	2300± 14 a	1239 ± 12 g	338 ± 11 f	51.5 ± 4 cd
	400	8680 ± 8 c	362± 12 fg	2150 ± 16 b	1474 ± 25 f	370 ± 7 e	58 ± 5 bc
	600	7969 ± 10 d	372 ± 8 f	1925± 28 d	1670± 20 d	375 ± 8 e	66.5 ± 4 b
***S. fruticosa***	0	6420 ± 10 f	438± 11 d	1578 ± 4 e	330± 16 l	337 ± 11 f	13.5 ± 0.75 i
	100	5760 ± 11 g	428± 11 d	1291 ± 35 f	811 ± 38 i	294 ± 11 g	31 ± 3 fg
	200	5028 ± 15 j	264 ± 9 h	1127 ± 28 h	1006 ± 19 h	468 ± 9 cd	40 ± 5 ef
	400	4217 ± 15 k	211 ± 8 i	866± 35 i	1560 ± 22 e	397 ± 7 e	55 ± 3 bcd
	600	8913 ± 12 b	262 ± 9 h	2075 ± 28 c	1782 ± 22 c	296 ± 8 g	90 ± 2.5 a
***A. macrostachyum***	0	3130 ±22 l	414 ± 13 de	85 ± 7 k	537± 22 j	454 ± 11 d	16.5 ± 1 hi
	100	5057 ± 8 i	494 ± 9 c	1162 ± 7 g	1202 ± 15 g	671 ± 11 a	25.75 ± 0.75 gh
	200	5736 ± 17 h	336 ± 8 g	1239 ± 7 g	1570± 7 e	496 ± 7 c	46.5 ± 2.75 de
	400	7760 ± 12 de	367 ± 8 f	1664± 10 d	2338 ± 17 b	607 ± 11 b	56 ± 1.25 bcd
	600	10652 ± 25 a	276 ± 11 h	2187 ± 7 b	2693 ± 13 a	620 ± 9 b	64 ± 3 b

**Table 2:** Two-way ANOVA of salinity, species, and their interaction on all tested parameters.

Parameters	Species	Species × Salinity	Salinity
shoot Fresh weight	***	***	***
shoot dry weight	***	***	***
Root fresh weight	***	***	***
Root Dry weight	***	***	***
Chl a	***	***	***
Chl b	***	***	***
Carotenoids	***	***	***
Chl a/b	***	***	**
MDA	***	***	***
Proline	***	***	***
phenol	***	***	***
Flavonoids	***	***	***

**: *p* ˂ 0.01 and ***: *p* ˂ 0.001.

## Data Availability

Not applicable.

## Supplementary Materials

The following are available online at https://www.mdpi.com/article/10.3390/plants10061100/s1, Figure S1: Superoxide dismutase isozymes of *S. europaea, S. fruticosa* and *A. macrostachyum* under different NaCl concentrations, Table S1: The correlation coefficient of growth parameters (shoot fresh weight: SFW, root fresh weight: RFW, shoot dry weight: SDW, root dry weight: RDW), Chl a, Chl b, Chl a/b, carotenoids: Car, Proline: pro, malondialdehyde: MDA, phenolics: Phe, Flavonoids: Flav. in *Arthrocnemum macrostachyum*, *Sarcocornia fruticosa* and *Salicornia europaea* under salinity treatments.
