# Supplementary material for: Differential Salt Tolerance Strategies in Three Halophytes from the Same Ecological Habitat: Augmentation of Antioxidant Enzymes and Compounds
Source: Plants (Basel). 2021 May 30;10(6):1100. doi: 10.3390/plants10061100 (PMC8229423; doi:10.3390/plants10061100)
Supplement: Supplementary file 1 [file plants-10-01100-s001.zip › plants-1207187-supplementary.pdf]

plants

MDPI

Supplementary Material

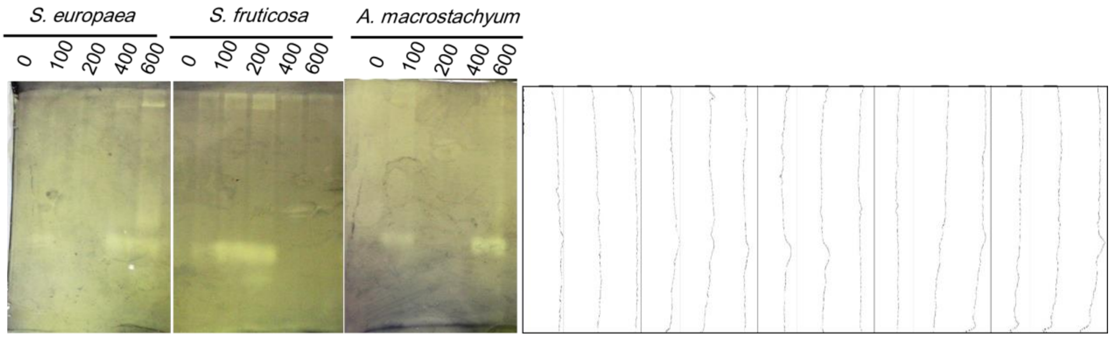

Figure S1. Superoxide dismutase isozymes of *S. europaea, S. fruticosa* and *A. macrostachyum* under different NaCl concentrations.

Table S1. The correlation coefficient of growth parameters (shoot fresh weight: SFW, root fresh weight: RFW, shoot dry weight: SDW, root dry weight: RDW), Chl a, Chl b, Chl a/b, carotenoids: Car, Proline: pro, malondialdehyde: MDA, phenolics: Phe, Flavonoids: Flav. in *Arthrocnemum macrostachyum*, *Sarcocornia fruticosa* and *Salicornia europaea* under salinity treatments.

	SFW	RFW	SDW	RDW	Chla	Chlb	Chlab	Car	Pro	MDA	Phe	Flav
						Arthrocnemum macrostachyum						
SFW	1	0.864 **	0.965 **	0.961 **	0.029	0.433	−0.466	−0.837 **	−0.824 **	−0.357	−0.748 **	−0.844 **
RFW	0.864 **	1	0.837 **	0.862 **	−0.068	0.667 **	−0.723 **	−0.893 **	−0.908 **	−0.724 **	−0.930 **	−0.950 **
SDW	0.965 **	0.837 **	1	0.969 **	−0.033	0.386	−0.450	−0.796 **	−0.778 **	−0.302	−0.741 **	−0.832 **
RDW	0.961 **	0.862 **	0.969 **	1	−0.028	0.484	−0.548 *	−0.819 **	−0.824 **	−0.358	−0.783 **	−0.848 **
Chla	0.029	−0.068	−0.033	−0.028	1	0.283	0.130	−0.213	−0.202	−0.166	−0.007	0.146
Chlb	0.433	0.667 **	0.386	0.484	0.283	1	−0.910 **	−0.704 **	−0.824 **	−0.820 **	−0.792 **	−0.695 **
Chlab	−0.466	−0.723 **	−0.450	−0.548 *	0.130	−0.910 **	1	0.641 **	0.777 **	0.735 **	0.820 **	0.795 **
Car	−0.837 **	−0.893 **	−0.796 **	−0.819 **	−0.213	−0.704 **	0.641 **	1	0.943 **	0.711 **	0.848 **	0.832 **
Pro	−0.824 **	−0.908 **	−0.778 **	−0.824 **	−0.202	−0.824 **	0.777 **	0.943 **	1	0.749 **	0.906 **	0.859 **
MDA	−0.357	−0.724 **	−0.302	−0.358	−0.166	−0.820 **	0.735 **	0.711 **	0.749 **	1	0.778 **	0.636 *
Phe	−0.748 **	−0.930 **	−0.741 **	−0.783 **	−0.007	−0.792 **	0.820 **	0.848 **	0.906 **	0.778 **	1	0.891 **
Flav	−0.844 **	−0.950 **	−0.832 **	−0.848 **	0.146	−0.695 **	0.795 **	0.832 **	0.859 **	0.636 *	0.891 **	1
						Sarcocornia fruticosa						
SFW	1	0.958 **	0.972 **	0.863 **	0.338	0.022	0.174	−0.806 **	−0.636 *	−0.710 **	−0.094	−0.555 *
RFW	0.958 **	1	0.980 **	0.942 **	0.423	0.218	0.023	−0.887 **	−0.761 **	−0.812 **	−0.197	−0.469
SDW	0.972 **	0.980 **	1	0.923 **	0.439	0.078	0.153	−0.840 **	−0.704 **	−0.726 **	−0.102	−0.609 *
RDW	0.863 **	0.942 **	0.923 **	1	0.539 *	0.302	−0.016	−0.888 **	−811 **	−0.750 **	−0.042	−0.482
Chla	0.338	0.423	0.439	0.539 *	1	0.092	0.378	−0.333	−0.383	−0.220	−0.026	−0.506
Chlb	0.022	0.218	0.078	0.302	0.092	1	−0.878 **	−0.448	−0.713 **	−0.650 **	−0.207	0.473
Chlab	0.174	0.023	0.153	−0.016	0.378	−0.878 **	1	0.218	0.482	0.453	0.180	−0.649 **
Car	−0.806 **	−0.887 **	−0.840 **	−0.888 **	−0.333	−0.448	0.218	1	0.847 **	0.888 **	0.138	0.170
Pro	−0.636 *	−0.761**	−0.704 **	−0.811 **	−0.383	−0.713 **	0.482	0.847 **	1	0.879 **	0.163	0.121
MDA	−0.710 **	−0.812 **	−0.726 **	−0.750 **	−0.220	−0.650 **	0.453	0.888 **	0.879 **	1	0.245	−0.017
Phe	−0.094	−0.197	−0.102	−0.042	−0.026	−0.207	0.180	0.138	0.163	0.245	1	−0.278
Flav	−0.555 *	−0.469	−0.609 *	−0.482	−0.506	0.473	−0.649 **	0.170	0.121	−0.017	−0.278	1
						Salicornia europaea						
SFW	1	0.618 *	0.833 **	0.874 **	0.712 **	0.687 **	−0.098	0.507	−0.903 **	−0.131	−0.047	−0.110

RFW	0.618 *	1	0.555 *	0.718 **	0.643 **	0.158	0.512	0.819 **	−0.686 **	0.397	−0.272	0.608 *
SDW	0.833 **	0.555 *	1	0.854 **	0.699 **	0.576 *	0.019	0.443	−0.822 **	−0.037	0.039	0.098
RDW	0.874 **	0.718 **	0.854 **	1	0.845 **	0.646 **	0.122	0.646 **	−0.937 **	0.112	−0.006	0.195
Chla	0.712 **	0.643 **	0.699 **	0.845 **	1	0.664 **	0.306	0.731 **	−0.885 **	0.192	−0.130	0.242
Chlb	0.687 **	0.158	0.576 *	0.646 **	0.664 **	1	−0.504	0.280	−0.709 **	−0.005	−0.091	−0.342
Chlab	−0.098	0.512	0.019	0.122	0.306	−0.504	1	0.489	−0.095	0.265	−0.064	0.732 **
Car	0.507	0.819 **	0.443	0.646 **	0.731 **	0.280	0.489	1	−0.754 **	0.638 *	−0.639 *	0.602*
Pro	−0.903 **	−0.686 **	−0.822 **	−0.937 **	−0.885 **	−0.709 **	−0.095	−0.754 **	1	−0.178	0.254	−0.132
MDA	−0.131	0.397	−0.037	0.112	0.192	−0.005	0.265	0.638 *	−0.178	1	−0.670 **	0.664 **
Phe	−0.047	−0.272	0.039	−0.006	−0.130	−0.091	−0.064	−0.639 *	0.254	−0.670 **	1	−0.331
Flav	−0.110	0.608 *	0.098	0.195	0.242	−0.342	0.732 **	0.602 *	−0.132	0.664 **	−0.331	1

* Correlation is significant at the 0.05 level (2−tailed). ** Correlation is significant at the 0.01 level (2−tailed).
